# Cold atmospheric plasma improves antifungal responsiveness of *Aspergillus flavus* and *Fusarium keratoplasticum* conidia and mycelia

**DOI:** 10.1371/journal.pone.0326940

**Published:** 2025-08-11

**Authors:** Darby M. Roberts, Jonathan E. Thomas, Jacklyn H. Salmon, Marc A. Cubeta, Katharina Stapelmann, Brian C. Gilger

**Affiliations:** 1 Departement of Clinical Sciences, College of Veterinary Medicine, North Carolina State University, Raleigh, North Carolina, United States of America; 2 Department of Nuclear Engineering, North Carolina State University, Raleigh, North Carolina, United States of America; 3 Department of Entomology and Plant Pathology, North Carolina State University, Raleigh, Center for Integrated Fungal Research, North Carolina, United States of America; University of California Riverside, UNITED STATES OF AMERICA

## Abstract

The purpose of this study is to evaluate sublethal cold atmospheric plasma (CAP) treatment of filamentous fungal pathogen susceptibility to commonly used antifungal drugs *in vitro*. Response to CAP in combination with voriconazole, fluconazole, amphotericin B, and caspofungin was evaluated in *Aspergillus flavus* and *Fusarium keratoplasticum* conidia and mycelium; conidial response to fluconazole was also assessed in three strains of *F. falciforme*. Conidial susceptibility to antifungal drugs alone or in combination with CAP was assessed using a modified CLSI broth microdilution assay with MIC determination and colony-forming unit (CFU) enumeration. Mycelial viability and biofilm thickness changes in response to antifungal drugs alone or in combination with CAP were assessed over 24 hours post treatment. CAP enhanced antifungal drug efficacy against all fungal species, though effects differed by drug and growth form. CAP enhanced antifungal drug susceptibility in conidia, with the strongest effect observed for *F. keratoplasticum* conidia, where caspofungin MIC decreased fourfold (from 16 to 4−8 μg/mL) and sensitivity to fluconazole, which exerted no effect in absence of CAP, was restored when combined with sublethal CAP treatment. In *A. flavus*, CAP lowered the MIC of voriconazole (from 0.25 to 0.06–0.125 μg/mL) but increased the MIC of amphotericin B (from 4 to >4 μg/mL), despite reductions in viable cell counts. Differential responses to CAP and fluconazole were observed across three strains of *F. falciforme*, suggesting variability in CAP response. In treated biofilms, CAP alone initially reduced mycelial viability and biofilm thickness, but partial recovery of the fungus was observed over time in most cases. When combined with antifungal drugs, CAP significantly enhanced reduction of mycelial viability and thickness beyond antifungal treatment alone. In *F. keratoplasticum* biofilms, the combination of CAP and antifungal drugs produced sustained reductions in mycelial viability and biofilm thickness, whereas *A. flavus* biofilms were more resistant to CAP treatment and exhibited consistent recovery after 24 hours.

## 1. Introduction

Fungal keratitis is a serious ocular disease associated with severe pain, decreased vision, and permanent blindness. It is estimated to affect over 2 million individuals annually, with its prevalence expected to rise due to an increasing population of at-risk individuals and the effects of climate change, which facilitate the spread and adaptation of causative pathogens [1–3]. *Aspergillus* and *Fusarium* species have been identified as leading causes of fungal keratitis [2,4]. Fungal conidia from the environment are introduced into the cornea through a break in the epithelial barrier, often caused by trauma from vegetative matter or contact lens misuse. Once in the cornea, conidia (asexual spores) germinate to establish a local invasive infection. Intracorneal germination of conidia results in extensive tissue damage and triggers a host immune response, further exacerbating tissue destruction [5]. Treatment of fungal keratitis typically involves an extended course of weeks to months of topical antifungal medication. Selection of antifungal medication is severely limited, with only three antifungal agents – voriconazole, amphotericin B, and natamycin – representing the most relied upon medications [6]. The limited arsenal of antifungal agents presents a significant management challenge for infections caused by *Aspergillus* and *Fusarium* species, a concern exacerbated by increasing reports of antifungal drug resistance in recent years [7,8]. In addition, biofilm formation introduces a complicating factor in cases of *Aspergillus* and *Fusarium* fungal keratitis, enhancing pathogenicity, virulence, and resistance to antifungal treatments [9–11].

Given the limitations of antifungal treatments and propensity for resistance, alternative approaches like photoactivated chromophore for corneal cross-linking (PACK-CXL) have been explored. PACK-CXL aims to inactivate microbes by employing a photo chromophore, such as riboflavin, which absorbs UV light, triggering a reaction that generates reactive oxygen species (ROS), including singlet oxygen, hydroxyl radicals, and hydrogen peroxide. However, there is limited evidence for the clinical benefit from incorporating PACK-CXL into fungal keratitis treatment strategies [12,13]. Reports of negative effects of PACK-CXL, such as ulcer deterioration and enhanced risk for infectious keratitis have further hindered application of this treatment [14–16].

An alternative approach, cold atmospheric plasma (CAP), generates ROS through non-thermal plasma without an activating light source or a chromophore. CAP has garnered extensive attention for its dual ability to inactivate a diverse array of multidrug-resistant microorganisms while stimulating cell proliferation to support wound healing [17–19]. This method has been shown to inactivate fungal keratitis pathogens *in vitro* and *in vivo* and has accelerated healing in clinical cases of fungal keratitis without reported negative effects [20–25]. Previously, we demonstrated that CAP effectively inactivates conidia and biofilms of the filamentous fungal keratitis pathogens, *Aspergillus flavus* and *Fusarium keratoplasticum* [21].

In addition to independently inactivating antimicrobial-resistant microorganisms, sublethal CAP treatment has also been shown to enhance antimicrobial susceptibility. Prior studies have demonstrated that sublethal CAP reduces MICs of antifungal and antibacterial agents in both biofilm and planktonic models, with effects observed in *Candida* spp. and *Pseudomonas aeruginosa* [22,26]. Notably, CAP has been reported to dramatically lower biofilm eradication thresholds when combined with antibiotics [22]. However, the impact of CAP on antifungal response in filamentous fungi remains unexplored.

Considering this knowledge gap, we investigated the antifungal response in filamentous fungal keratitis pathogens to CAP treatment. The primary objective of this study is to determine if applying the minimum effective dose of CAP, as previously described, to *A. flavus* and *F. keratoplasticum* conidia and biofilms before treating with an antifungal drug enhances the antifungal response [21]. We hypothesized there would be drug- and fungal species-dependent changes to antifungal response following sublethal CAP treatment. A modified CLSI broth microdilution assay was used to assess fungal conidial response to the minimum effective dose of CAP combined with a range of antifungal concentrations. In contrast, biofilm response was characterized using metabolic activity measurements, biofilm thickness analysis, and microscopic morphological evaluation following treatment with the minimum effective dose of CAP and a single antifungal concentration corresponding to the known conidial MIC. Voriconazole, amphotericin B, and caspofungin were included in this study to represent three widely used antifungal drug classes: azoles, polyenes, echinocandins, respectively, while fluconazole was selected to represent an antifungal drug to which both fungal species included in the study have demonstrated resistance against [27]. We additionally investigate the time-kill kinetics of *A. flavus* and *F. keratoplasticum* CAP-treated biofilms and effect of sublethal CAP treatment on biofilm eradication alone and in combination with antifungal drugs.

## 2. Methods

### 2.1. Fungal conidial suspension and biofilm preparation

The *Aspergillus flavus*, *Fusarium keratoplasticum*, and *F. falciforme* strains used in this study were cryopreserved clinical isolates originally collected, purified, and identified as previously described [27]. The *A. flavus* isolate used for this study was identified as Multi-locus Sequence Type (MLST) designation AF9 lineage subgroup IC, while the *F. keratoplasticum* isolate was identified as MLST designation FK1 haplotype 2u [27]. The *F. falciforme* isolates were identified as MLST designations FF6 (IC22918), FF7 (IC22919), and FF8 (IC22927), all belonging to haplotype 4hhhh, 4ffff [27].

Each species was recovered from −80°C on Potato Dextrose Agar (PDA, Difco, Franklin Lakes, NJ, USA) at 33°C, 5% CO2 for 3–5 days. Conidial suspensions were made by flooding each plate with 3–5 ml potato dextrose broth prepared at a 50% dilution (PDB50, Difco, Franklin Lakes, NJ, USA). The agar surface was scraped using a sterile rubber spatula, and the resulting suspension was filtered through sterile cheesecloth lining a funnel. The suspension was diluted 1:10 with PDB50, and concentration was adjusted to 1 × 10^5^ conidia per ml with a hemocytometer (Hausser Scientific, Horsham, PA, USA.

Biofilms were prepared by pipetting 150 μL of conidial suspension and 450 μL of PDB50 into select wells of sterile 24-well flat-bottom tissue culture plates (Corning Inc., Corning, NY, USA). Plates were incubated at 33°C, 5% CO2 for 24 h. After incubation, the medium was aspirated, and biofilms were washed three times with 1–2 ml of phosphate-buffered saline (PBS, Thermo Fischer Scientific, Waltham, MA, USA) to remove unattached cells.

### 2.2. Cold atmospheric plasma treatment

CAP treatment was performed using a dielectric barrier discharge device with a cylindrical copper electrode covered by an aluminum oxide dielectric. Full device specifications are available in references [21]. Treatment parameters were set at 22 kV, 20 mA, and 300 Hz for all experiments.

For treatment of conidial suspensions before antifungal response testing, CAP treatment was applied to *A. flavus* and *F. keratoplasticum* by adding 150 µL of 1 × 10⁵ conidia/mL suspension and 150 µL of PDB50 to each well of a 24-well plate. An aluminum grounding plate was fitted to the bottom of the culture plate, and the DBD electrode was positioned over the center of the sample well, maintaining a 1.5-mm gap between the liquid surface and electrode tip.

For biofilm CAP treatments, prewashed *A. flavus* and *F. keratoplasticum* biofilms were treated under similar conditions. An aluminum grounding plate was placed beneath the culture plate, and the DBD electrode was positioned over the center of the sample well with a 1-mm gap between the sample surface and electrode tip.

The minimum effective treatment duration was applied for each species to accomplish sublethal CAP treatment. Sublethal treatment was defined as the minimum CAP exposure that produced a statistically significant reduction in viability without complete inactivation. The specific viability reductions used to define sublethal exposure were: ≤ 90% reduction in metabolic activity and ≤55% reduction in CFU/mL for ***A. flavus*** conidia; ≤ 30% metabolic reduction and ≤50% CFU/mL reduction for ***F. keratoplasticum*** conidia; ≤ 30% reduction in metabolic activity for ***A. flavus*** and ***F. keratoplasticum*** biofilms; and ≤70% and ≤45% CFU/mL reduction for ***A. flavus*** and ***F. keratoplasticum*** biofilms, respectively [21]. CAP treatment parameters were 22 kV, 20 mA, and 300 Hz, using a dielectric barrier discharge system as previously described, with minimum effective treatment durations of 4 min for ***A. flavus*** conidia, 3 min for ***F. keratoplasticum*** and ***F. falciforme*** conidia, and 1 min for biofilms of both species [21].

### 2.3. Antifungal susceptibility assay

A modified CLSI antifungal response assay was used to assess the effect of CAP treatment on *A. flavus* and *F. keratoplasticum* susceptibility to voriconazole (VOR), fluconazole (FLU), amphotericin B (AMB), and caspofungin (CAS), as well as *F. falciforme* susceptibility to FLU [28]. Stock solutions of 25 mg/mL of each drug were prepared by dissolving 25 mg of dry analytical-grade drug in dimethyl sulfoxide (DMSO, Sigma-Aldrich, St. Louis, MO, USA) and stored at −20°C. Further dilutions were prepared as needed with PDB50. For all drugs, the highest drug concentration was set at a previously determined minimum inhibitory concentration (MIC), while the lowest was 1/32 of the MIC, with stepwise half-fold dilutions in between [29]. Where published MIC data for the strains used in this study was not available, the MIC was determined using a standard CLSI antifungal response assay prior to further assays. For *A. flavus*, the concentration range of VOR was 0.0078 to 0.25 µg/mL, FLU was 4.875–156 µg/mL, AMB was 0.125–4 µg/mL, and CAS was 4.875–156 µg/mL. For *F. keratoplasticum*, the concentration range of VOR was 0.25–8 µg/mL, FLU was 4.875–156 µg/mL, AMB was 4.875 µg/mL, and CAS was 0.5–16 µg/mL. A FLU range of 4.875–156 µg/mL was used for *F. falciforme*.

Three hundred microliters of serially diluted drug solutions (prepared at 2X target concentration) were added to wells of a 24-well plate, each containing 150 µL of conidial suspension and 150 µL of PDB50. Each drug concentration was tested in triplicate in CAP-treated wells and in triplicate in non-CAP-treated wells. Wells receiving no antifungal drug served as growth controls and wells receiving PDB50 only served as sterile controls. Each experiment was performed twice on different days to ensure reproducibility. The MIC readings were determined at 72 h post-incubation at 33°C, 5% CO₂. Wells selected for the MIC displayed 100% visual growth inhibition.

To further assess the effects of CAP treatment on fungal conidial response to antifungal drugs, colony-forming unit enumeration was performed after each MIC assay. The contents of each well were thoroughly mechanically disrupted with a 1000 μL pipette tip and the well contents were transferred to a 2 ml round bottom Eppendorf tube and vortexed. All wells were washed with 600 μL PDB50 and the well contents were transferred to the corresponding Eppendorf tube. The resultant suspensions were serially diluted with PDB50. Aliquots (20 μL) of appropriate dilutions and undiluted samples, as well as uninoculated PDB50 (negative control), were plated onto PDA and incubated at 33°C, 5% CO2 for 24–48 h. The number of fungal colonies was counted and reported as CFU/mL.

### 2.4. Biofilm viability and thickness assays

The effect of sublethal CAP treatment on antifungal response was assessed in mature *A. flavus* and *F. keratoplasticum* biofilms exposed to VOR, FLU, AMB, and CAS. Twenty-four hours post-inoculation, pre-washed biofilms were divided into CAP-treated and non-CAP-treated groups. Within each group, three biofilms were assigned to each antifungal treatment. Following CAP treatment of selected wells, 450 μL of antifungal solution prepared at the conidial MIC was added to the designated wells. Mycelial viability and biofilm thickness were assessed at 0, 3, 6, 9, 12, and 24 h post-CAP treatment, with three biofilms analyzed per condition at each time point. All imaging was performed with an Olympus IX83 inverted microscope outfitted with an Olympus DP80 dual-CCD digital camera. Images were acquired using OLYMPUS cellSense Dimension 4.2.1 imaging software.

Mycelial viability was assessed using a modified XTT ((2,3-bis-(2-methoxy-4-nitro-5-sulfophenyl)-2H-tetrazolium-5-carboxanilide, disodium salt) reduction assay [30]. At each sampling time, wells were aspirated and washed with 500 μL PBS to remove nonadherent cells before adding 200 μL of XTT solution (200 μg/mL; Thermo Fischer Scientific) supplemented with 25 μM menadione (MilliporeSigma, Burlington, MA, USA). Wells were incubated at 33°C with 5% CO₂ for 3 h. Following incubation, the supernatant was removed, and phase-contrast images of the central well area were obtained in the RGB system with a 4X/0.13 NA lens for a total magnification of 2.56 X. The central position of each well in the 24-well plate was pre-mapped using cellSense plate mapping software to ensure consistency in imaging. XTT reduction was quantified using ImageJ (Fiji, version 1.52k) software. A color threshold specific to XTT reduction (Hue: 0–50, Saturation: 30–255, Brightness: 0–255) was determined and applied to each image using the ImageJ “Image → Adjust → Color Threshold” function. The gated area was quantified using the “Analyze → Analyze Particles” function and reported as viable mycelial area in pixel count.

Biofilm thickness was quantified using phase-contrast microscopy by determining the focal positions of the biofilm’s bottom and top surfaces. This approach is adapted from previously established methods for biofilm thickness measurement [31]. An inverted microscope equipped with a motorized fine-focus adjustment knob was used with a 40X/0.6 NA lens for a total magnification of 25.2 X. The focal plane was first adjusted to focus on the lowest visible biofilm layer, corresponding to the attachment interface between the biofilm and well bottom. The focal plane was raised until the biofilm’s uppermost layer came into focus. The vertical distance between these two focal positions was recorded as biofilm thickness. Measurements were taken at the center of each well, with three biofilms analyzed per condition per time point.

Brightfield microscopy was performed on representative CAP-treated and untreated *A. flavus* and *F. keratoplasticum* mycelium 24 h post-treatment for morphology assessment. Brightfield grayscale images of the central biofilm area were obtained with a 40X/0.6 NA lens for a total magnification of 25.2 X.

### 2.5. Statistical analysis

**Fig 1:**
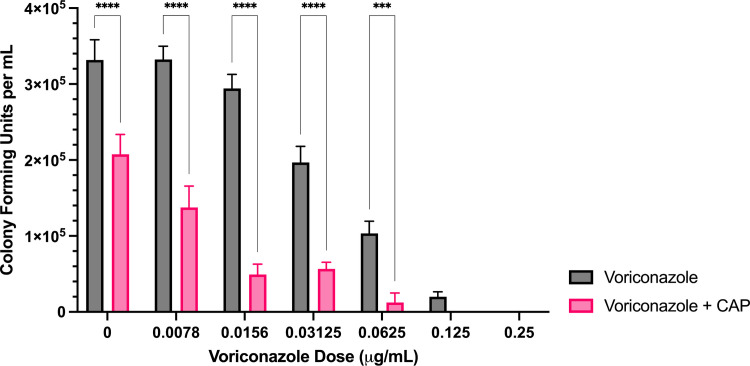
Sublethal CAP treatment enhances *A. flavus* voriconazole sensitivity. Colony forming unit enumeration of *A. flavus* conidia treated with voriconazole, CAP alone, or a combination of voriconazole and cold atmospheric plasma (CAP; 22 kV, 240 s) following 72 h incubation post-exposure. Significant differences relative to the non- antifungal drug control (0 µg/mL) within each treatment group (Voriconazole and Voriconazole + CAP) are indicated, as well as significant differences between treatment groups at each antifungal drug concentration; ***p < 0.001, ****p < 0.0001 (mean ± SEM; n = 6).

**Fig 2:**
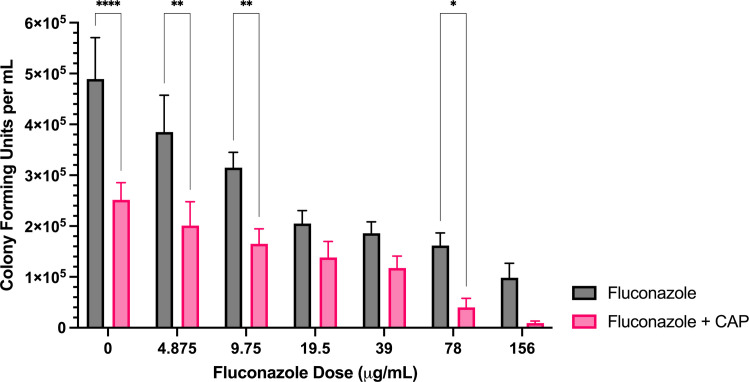
Sublethal CAP treatment enhances *A. flavus* fluconazole sensitivity. Colony forming unit enumeration of *A. flavus* conidia treated with fluconazole, CAP alone, or a combination of fluconazole and cold atmospheric plasma (CAP; 22 kV, 240 s) following 72 h incubation post-exposure. Significant differences relative to the non-antifungal drug control (0 µg/mL) within each treatment group (Fluconazole and Fluconazole + CAP) are indicated, as well as significant differences between treatment groups at each antifungal drug concentration; *p < 0.05, **p < 0.01,***p < 0.001, ****p < 0.0001 (mean ± SEM; n = 6).

**Fig 3:**
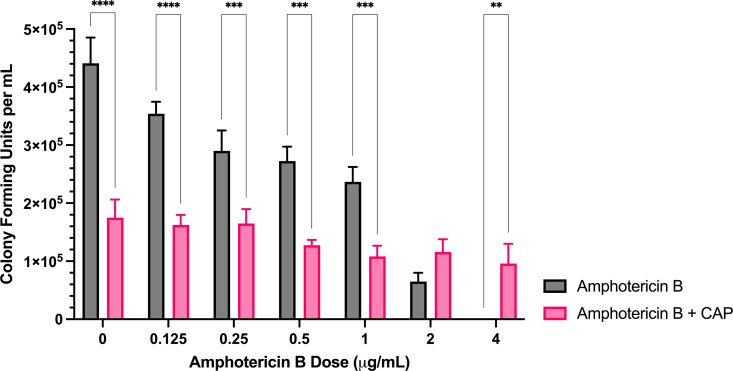
Sublethal CAP treatment restores *A. flavus* colony formation in presence of high dose amphotericin B. Colony forming unit enumeration of *A. flavus* conidia treated with amphotericin B, CAP alone, or a combination of amphotericin B and cold atmospheric plasma (CAP; 22 kV, 240 s) following 72 h incubation post-exposure. Significant differences relative to the no-drug control (0 µg/mL) within each treatment group (Amphotericin B and Amphotericin B + CAP) are indicated, as well as significant differences between treatment groups at each antifungal drug concentration; **p < 0.01,***p < 0.001, ****p < 0.0001 (mean ± SEM; n = 6).

**Fig 4:**
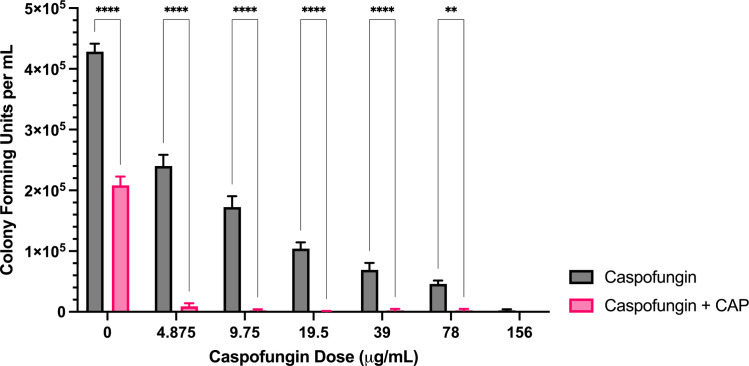
Sublethal CAP treatment markedly enhances *A. flavus* caspofungin sensitivity. Colony forming unit enumeration of *A. flavus* conidia treated with caspofungin, CAP alone, or a combination of caspofungin and cold atmospheric plasma (CAP; 22 kV, 240 s) following 72 h incubation post-exposure. Significant differences relative to the non-antifungal drug control (0 µg/mL) within each treatment group (Caspofungin and Caspofungin + CAP) are indicated, as well as significant differences between treatment groups at each antifungal drug concentration; **p < 0.01, ****p < 0.0001 (mean ± SEM; n = 6).

**Fig 5:**
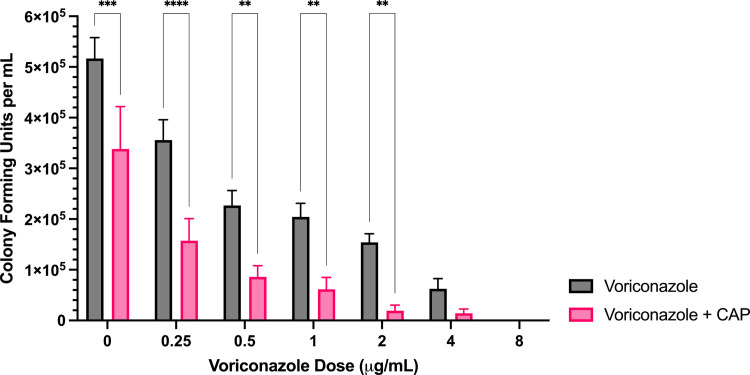
Sublethal CAP treatment enhances *F. keratoplasticum* voriconazole B sensitivity. Colony forming unit enumeration of *F. keratoplasticum* conidia treated with voriconazole, CAP alone, or a combination of voriconazole and cold atmospheric plasma (CAP; 22 kV, 180 s) following 72 h incubation post-exposure. Significant differences relative to the non-antifungal drug control (0 µg/mL) within each treatment group (Voriconazole and Voriconazole + CAP) are indicated, as well as significant differences between treatment groups at each antifungal drug concentration; *p < 0.05, **p < 0.01,***p < 0.001, ****p < 0.0001 (mean ± SEM; n = 6).

**Fig 6:**
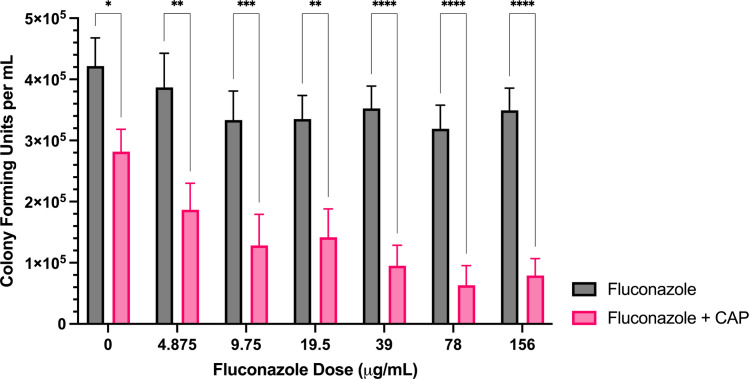
Sublethal CAP treatment restores *F. keratoplasticum* fluconazole sensitivity. Colony forming unit enumeration of *F. keratoplasticum* conidia treated with fluconazole, CAP alone, or a combination of fluconazole and cold atmospheric plasma (CAP; 22 kV, 180 s) following 72 h incubation post-exposure. Significant differences relative to the non-antifungal drug control (0 µg/mL) within each treatment group (Fluconazole and Fluconazole + CAP) are indicated, as well as significant differences between treatment groups at each antifungal drug concentration; *p < 0.05, **p < 0.01,***p < 0.001, ****p < 0.0001 (mean ± SEM; n = 6).

**Fig 7:**
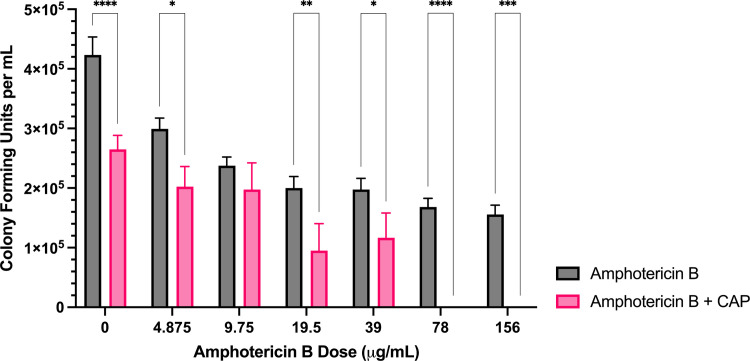
Sublethal CAP treatment enhances *F. keratoplasticum* amphotericin B sensitivity. Colony forming unit enumeration of *F. keratoplasticum* conidia treated with amphotericin B, CAP alone, or a combination of amphotericin B and cold atmospheric plasma (CAP; 22 kV, 180 s) following 72 h incubation post-exposure. Significant differences relative to the no-drug control (0 µg/mL) within each treatment group (Amphotericin B and Amphotericin B + CAP) are indicated, as well as significant differences between treatment groups at each antifungal drug concentration; *p < 0.05, **p < 0.01,***p < 0.001, ****p < 0.0001 (mean ± SEM; n = 6).

**Fig 8:**
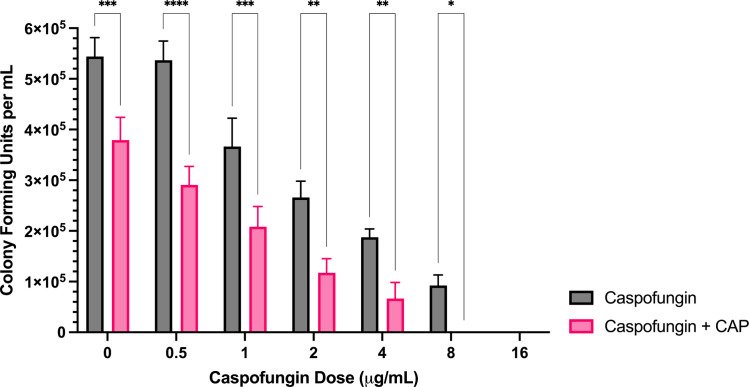
Sublethal CAP treatment enhances *F. keratoplasticum* caspofungin sensitivity. Colony forming unit enumeration of *F. keratoplasticum* conidia treated with caspofungin, CAP alone, or a combination of caspofungin and cold atmospheric plasma (CAP; 22 kV, 180 s) following 72 h incubation post-exposure. Significant differences relative to the non-antifungal drug control (0 µg/mL) within each treatment group (Caspofungin and Caspofungin + CAP) are indicated, as well as significant differences between treatment groups at each antifungal drug concentration; *p < 0.05, **p < 0.01,***p < 0.001, ****p < 0.0001 (mean ± SEM; n = 6).

**Fig 9:**
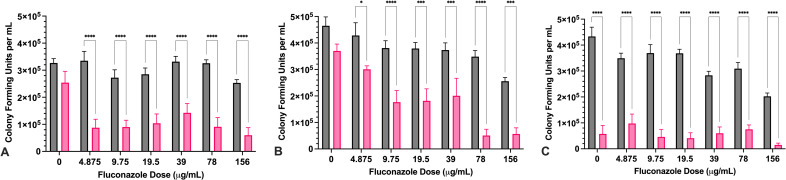
Sublethal CAP treatment differentially affects viability and fluconazole sensitivity of closely related strains of *F. falciforme* strains. Colony forming unit enumeration of three strains of *F. falciforme* conidia treated with fluconazole, CAP alone, or a combination of fluconazole and cold atmospheric plasma (CAP; 22 kV, 180 s) following 72 h incubation post-exposure. (A) IC22919; (B) IC22927; (C) IC22918. Significant differences relative to the non-antifungal drug control (0 µg/mL) within each treatment group (Fluconazole and Fluconazole + CAP) are indicated, as well as significant differences between treatment groups at each antifungal drug concentration; *p < 0.05, **p < 0.01,***p < 0.001, ****p < 0.0001 (mean ± SEM; n = 6).

**Fig 10:**
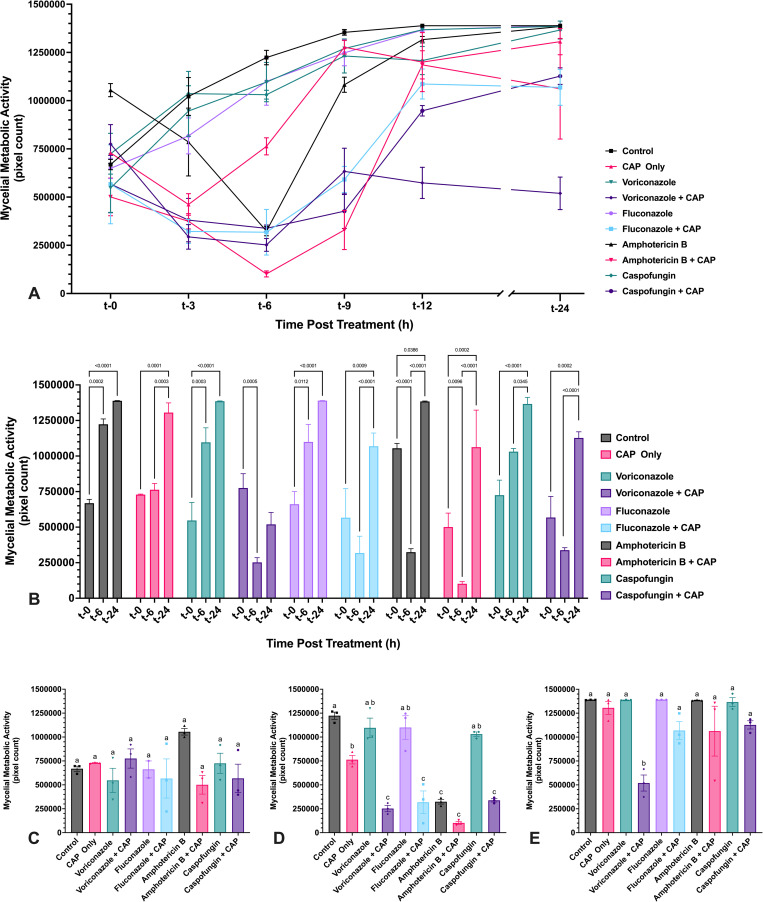
CAP treatment of *A. flavus* mycelium induces transient reduction in metabolic activity, which is extended in the presence of voriconazole. Metabolic activity of *A. flavus* mycelium following exposure to either voriconazole, fluconazole, amphotericin B, or caspofungin alone and in combination with cold atmospheric plasma (CAP; 22 kV, 60 s). (A) Metabolic activity of *A. flavus* mycelium was assessed at 24 h post-inoculation following treatment with antifungal drugs alone or in combination with CAP. Phase-contrast microscopic images were acquired at 0, 3, 6, 9, 12, and 24 h post treatment with antifungal drugs alone or in combination with CAP, and the area of metabolically active mycelium was quantified using pixel-based analysis. (B) Significant changes in metabolic activity within each treatment group over time (t-0, t-6, t-24) are indicated. (C–E) Significant differences in metabolic activity between treatment groups at (C) t-0, (D) t-6, and (E) t-24. Different lowercase letters represent significant differences between treatment groups (*p* < 0.05). Data represent mean ± SEM (n = 3).

**Fig 11:**
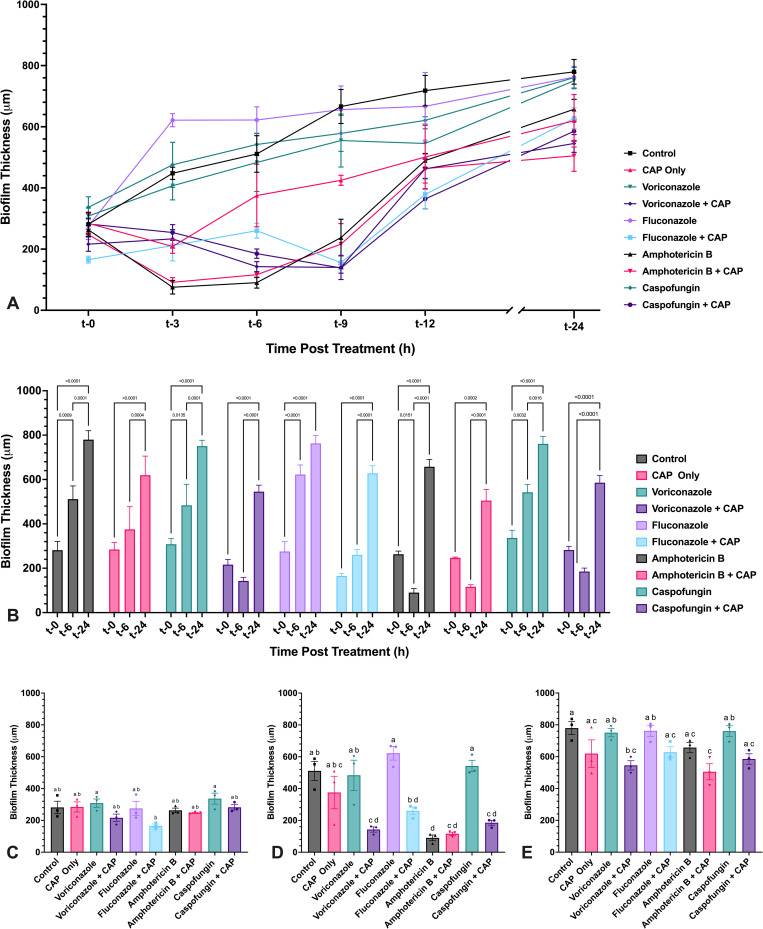
CAP treatment of *A. flavus* mycelium induces transient reduction in biofilm thickness when coupled with antifungal drug treatment. *A. flavus* biofilm thickness following exposure to either voriconazole, fluconazole, amphotericin B, or caspofungin alone and in combination with cold atmospheric plasma (CAP; 22 kV, 60 s). (A) *A. flavus* biofilms were treated at 24 h post-inoculation, and thickness was measured at 0, 3, 6, 9, 12, and 24 h post treatment with antifungal drugs alone or in combination with CAP. (B) Significant changes in biofilm thickness within each treatment group over time (t-0, t-6, t-24) are indicated. (C–E) Significant differences in biofilm thickness between treatment groups at (C) t-0, (D) t-6, and (E) t-24. Different lowercase letters represent significant differences between treatment groups (*p* < 0.05). Data represent mean ± SEM (n = 3).

**Fig 12:**
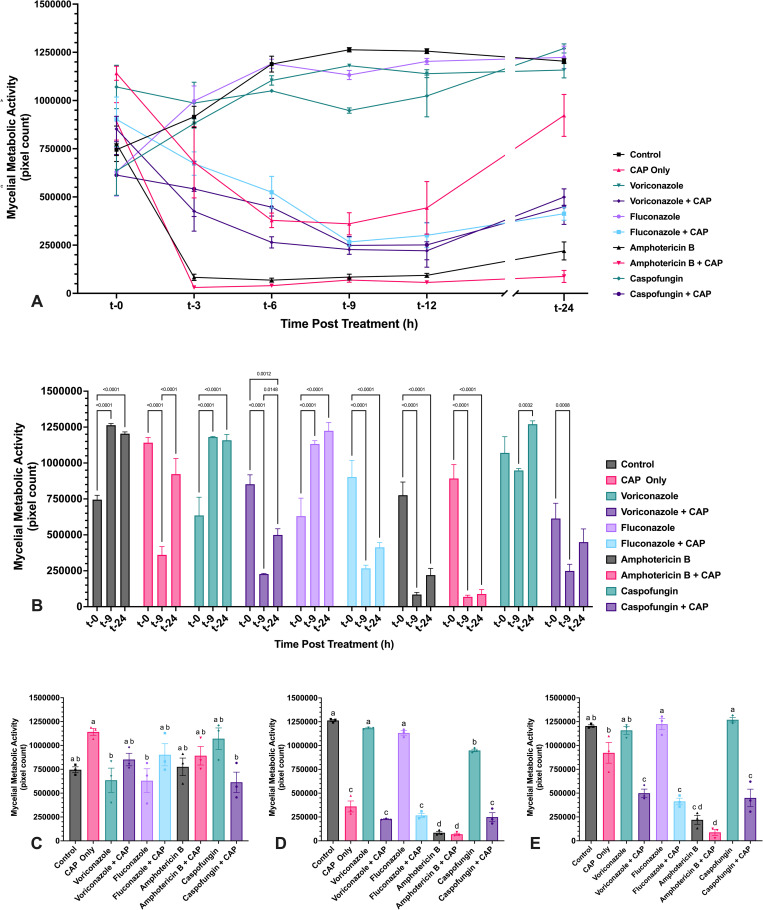
CAP treatment of *F. keratoplasticum* mycelium induces transient reduction in metabolic activity, which is extended in the presence of antifungal drugs. Metabolic activity of *F. keratoplasticum* mycelium following exposure to either voriconazole, fluconazole, amphotericin B, or caspofungin alone and in combination with cold atmospheric plasma (CAP; 22 kV, 60 s). (A) Metabolic activity of *F. keratoplasticum* mycelium was assessed at 24 h post-inoculation following treatment with antifungal drugs alone or in combination with CAP. Phase-contrast microscopic images were acquired at 0, 3, 6, 9, 12, and 24 h post treatment with antifungal drugs alone or in combination with CAP, and the area of metabolically active mycelium was quantified using pixel-based analysis. (B) Significant changes in metabolic activity within each treatment group over time (t-0, t-9, t-24) are indicated. (C–E) Significant differences in metabolic activity between treatment groups at (C) t-0, (D) t-9, and (E) t-24. Different lowercase letters represent significant differences between treatment groups (*p* < 0.05). Data represent mean ± SEM (n = 3).

**Fig 13:**
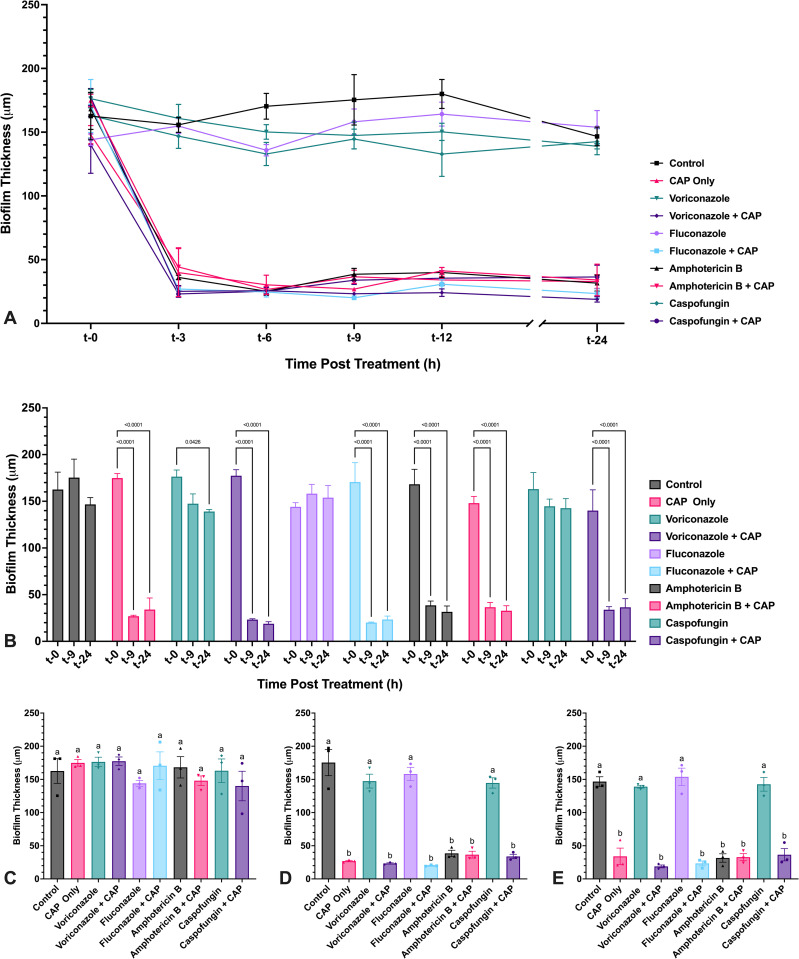
CAP treatment of *F. keratoplasticum* mycelia induces non-resolving reduction in biofilm thickness, alone and in the presence of antifungal drugs. *F. keratoplasticum* biofilm thickness following exposure to either voriconazole, fluconazole, amphotericin B, or caspofungin alone and in combination with cold atmospheric plasma (CAP; 22 kV, 60 s). (A) *F. keratoplasticum* biofilms were treated at 24 h post-inoculation, and thickness was measured at 0, 3, 6, 9, 12, and 24 h post treatment with antifungal drugs alone or in combination with CAP. (B) Significant changes in biofilm thickness within each treatment group over time (t-0, t-9, t-24) are indicated. (C–E) Significant differences in biofilm thickness between treatment groups at (C) t-0, (D) t-9, and (E) t-24. Different lowercase letters represent significant differences between treatment groups (*p* < 0.05). Data represent mean ± SEM (n = 3).

Two-way ANOVA with Šídák’s post-hoc analysis for multiple mean comparisons was performed to determine significant differences among treatment means of conidial colony forming units (Figs 1–9, S1 Table). Two-way ANOVA with Tukey’s post-hoc analysis for multiple means comparisons was performed to determine significant differences among means of mycelial metabolic activity and biofilm thickness within treatment groups (Figs 10–13B). Ordinary one-way ANOVA with Tukey’s post-hoc analysis for multiple means comparisons was performed to determine significant differences among means of mycelial metabolic activity and biofilm thickness across treatment groups at a single time point (Figs 10–13C–E). Differences were considered significant at P ≤ 0.05. All data analyses were performed using GraphPad Prism v10.4.1 for macOS (GraphPad Software, Boston, MA, USA).

## 3. Results

### 3.1. Pretreatment with sublethal cold atmospheric plasma reduces the MIC of select antifungal drugs for *Aspergillus flavus* and *Fusarium keratoplasticum*

The minimum inhibitory concentrations (MIC) of voriconazole, fluconazole, amphotericin B, and caspofungin for *A. flavus* and *F. keratoplasticum* are presented in Table 1. Cold atmospheric plasma (CAP) treatment reduced the MIC of voriconazole and fluconazole for *A. flavus* from 0.25 µg/mL to 0.125 µg/mL, and from >156 µg/mL to 156 to >156 µg/mL, respectively. However, the MIC of amphotericin B increased from 4 µg/mL to >4 µg/mL following CAP treatment. For *F. keratoplasticum* treated with CAP, the MIC of caspofungin decreased from 16 µg/mL to 4–8 µg/mL.

**Table 1:** Minimum inhibitory concentrations (µg/mL) of voriconazole, fluconazole, amphotericin B, and caspofungin for *Aspergillus flavus* and *Fusarium keratoplasticum*, with (treated) and without (untreated) sublethal cold atmospheric plasma exposure prior to introduction of antifungal drug.

Species	Voriconazole (μg/mL)		Fluconazole (μg/mL)		Amphotericin B (μg/mL)		Caspofungin (μg/mL)	
	Untreated	Treated	Untreated	Treated	Untreated	Treated	Untreated	Treated
*A. flavus*	0.25	**0.06 - 0.125**	>156	**156 - > 156**	4	**>4**	>156	>156
*F. keratoplasticum*	8	8	>156	>156	>156	**78**	16	**4 - 8**

^a^Bolded results indicate a change in MIC in the CAP treated group.

### 3.2. Cold atmospheric plasma enhances fungal conidial response to sublethal antifungal drug concentrations

#### 3.2.1. Effects of CAP pre-treatment on *A. flavus* antifungal response.

*Voriconazole:* The combined treatment of CAP and voriconazole significantly reduced colony formation per ml (CFU/mL) compared to voriconazole alone at concentrations ranging from 0–0.0625 μg/mL (Fig 1). At voriconazole concentrations of ≥ 0.0156 μg/mL, the reduction in colony formation relative to the untreated control was significantly greater with CAP + voriconazole than with CAP alone (S1 Table). Complete inhibition occurred at 0.25 μg/mL for voriconazole alone and at 0.125 μg/mL for the combined treatment.

*Fluconazole:* Fluconazole alone significantly reduced colony formation at concentrations of 9–156 μg/mL (Fig 2). In combination with CAP, reductions were observed at all tested concentrations; however, significant improvements over fluconazole alone were only observed at 0, 4.875, 9.75, and 78 μg/mL (Fig 2). At 78 and 156 μg/mL, the combined treatment of CAP with fluconazole led to significantly greater reductions in CFU/mL than CAP alone (S1 Table).

*Amphotericin B:* Combining CAP with amphotericin B significantly reduced colony formation at 0–1 μg/mL compared to amphotericin B alone (Fig 3). However, at 4 μg/mL, CFU/mL was significantly greater with the combination treatment than with amphotericin B alone.

*Caspofungin:* Caspofungin alone had an MIC > 156 μg/mL (Table 1). While CAP treatment did not lower the MIC, near complete inhibition of colony formation occurred at caspofungin concentrations as low as 4.875 μg/mL when combined with CAP (Fig 4). At all concentrations <156 μg/mL, CAP significantly enhanced reductions in colony formation compared to caspofungin alone.

#### 3.2.2. Effects of CAP pre-treatment on *F. keratoplasticum* antifungal response.

*Voriconazole*: The combined treatment of CAP with voriconazole significantly reduced colony formation compared to voriconazole alone at concentrations from 0–2 μg/mL (Fig 5). At all voriconazole concentrations, the reduction in CFU/mL relative to the untreated control was significantly greater with CAP + voriconazole than with CAP alone (S1 Table).

*Fluconazole: F. keratoplasticum* was insensitive to fluconazole (MIC > 156 μg/mL), with no reduction in colony formation compared to untreated controls at any concentration (S1 Table). However, the combination of CAP and fluconazole significantly reduced colony formation at all concentrations compared to the untreated control, with the greatest reduction (77%) observed at 156 μg/mL (Fig 6). At 39–156 μg/mL, combination treatment resulted in significantly greater CFU/mL reductions than CAP alone (S1 Table).

*Amphotericin B:* The combined treatment of CAP with amphotericin B significantly reduced colony formation compared to amphotericin B alone at all concentrations except 9.75 μg/mL (Fig 7). At concentrations of 19.5 μg/mL and above, the reduction was significantly greater than with CAP alone (S1 Table). Complete inhibition of colony formation occurred at 78 μg/mL with the combination treatment, whereas amphotericin B alone achieved a maximal 37% reduction at 156 μg/mL compared to the untreated control (Fig 7).

*Caspofungin:* At concentrations of 0–8 μg/mL, combination treatment with CAP significantly reduced colony formation compared to caspofungin alone (Fig 8). At concentrations ≥1 μg/mL, reductions exceeded those achieved with CAP alone (S1 Table).

#### 3.2.3. Effects of CAP pre-treatment on *F. falciforme* fluconazole response.

To further investigate observations of fluconazole-insensitive *Fusarium keratoplasticum*, we conducted experiments to evaluate whether a similar response to CAP and fluconazole occurs in a closely related species, *Fusarium falciforme*, a member of the *Fusarium solani* species complex (FSSC) and common fungal keratitis pathogen [2,27]. Specifically, our experimental objective was to examine three clinical isolates of *F. falciforme* (IC22918, IC22919, and IC22927) within the same species complex with a similar multi-locus sequence haplotype and a previously determined MIC of >156 μg/mL for fluconazole [27]. While MIC values indicated fluconazole insensitivity, we aimed to determine whether this classification was supported when assessing CFU/mL and to explore variability in CAP’s effects across closely related isolates. To address these aims, we performed antifungal susceptibility testing (AST) and CFU enumeration following treatment with or without CAP and fluconazole ranging in concentrations from 0–156 μg/mL).

Of the three *F. falciforme* strains tested, only IC22919 showed complete insensitivity to fluconazole, with no significant reduction in colony formation at any concentration (S1 Table). However, when combined with CAP, fluconazole significantly reduced colony formation at all tested concentrations compared to fluconazole alone (Fig 9A). This pattern of restored fluconazole activity following CAP treatment closely resembled the response observed in the *F. keratoplasticum* isolate.

IC22927 was sensitive to fluconazole alone only at the highest concentrations (78 and 156 μg/mL; S1 Table). Combining fluconazole with CAP significantly reduced colony formation compared to fluconazole alone at all tested concentrations (Fig 9B). This combination also outperformed CAP alone at fluconazole concentrations of 9.75 μg/mL and above (S1 Table).

IC22918 exhibited a high degree of sensitivity to CAP alone. CAP treatment reduced colony formation by an average of 3.5 × 10⁵ CFU/mL (84%) compared to the untreated controls (Fig 9C). No additional significant reduction was observed with fluconazole co-treatment (S1 Table). However, at all fluconazole concentrations tested, colony formation was significantly lower with the combination treatment than with fluconazole alone (Fig 9C).

### 3.3. Effects of cold atmospheric plasma and antifungal drug, alone or in combination, on biofilm viability and thickness of *Aspergillus flavus* and *Fusarium keratoplasticum*

#### 3.3.1. *A. flavus* viability and thickness changes.

Metabolic activity of mature *A. flavus* mycelium was measured at regular intervals over 24 h following sublethal CAP treatment using a modified XTT reduction assay (S1 Fig). At the same intervals, biofilm thickness was approximated by measuring hyphal depth in the treated area. At t-0, immediately following CAP treatment of select wells and before antifungal drug exposure, no significant differences were observed in metabolic activity or biofilm thickness between untreated controls and any treatment group (Figs 10C and 11C). Untreated mycelium and mycelium treated with voriconazole, fluconazole, or caspofungin alone exhibited a steady increase in metabolic activity, plateauing between t-9 and t-12 (Fig 10A). *A. flavus* biofilms also continued to increase in thickness throughout the 24 h assessment period (Fig 11A).

CAP-only treatment resulted in a transient suppression of metabolic activity, reaching maximal reduction by t-3 (Fig 10A). Metabolic recovery began by t-6 and was complete by t-24, with CAP-treated *A. flavus* mycelium becoming statistically indistinguishable from untreated controls (Fig 10B, D, E).

When CAP was combined with antifungal drugs, *A. flavus* metabolic suppression was initially more pronounced and prolonged compared to CAP alone, with the most substantial reductions observed at t-6 (Fig 10D). However, by t-24, metabolic activity in all combination treatment groups had either returned to baseline or exceeded initial levels of metabolic activity. Only voriconazole + CAP remained significantly reduced compared to the untreated controls at t-24 (Fig 10E).

#### 3.3.2. *F. keratoplasticum* biofilm viability and thickness changes.

Mycelial metabolic activity and biofilm thickness in *F. keratoplasticum* were also assessed following CAP treatment, both alone and in combination with antifungal drugs (S2 Fig). At t-0, no significant differences were observed in either metabolic activity or biofilm thickness between untreated controls and any treatment group (Figs 12C and 13C). However, metabolic activity of *F. keratoplasticum* mycelium immediately after CAP treatment was significantly elevated compared to select treatment groups (voriconazole + CAP, fluconazole + CAP, and caspofungin + CAP) suggesting a short-term stimulatory effect of CAP. This transient metabolic activation has been observed previously following sublethal CAP exposure [21].

Untreated mycelium (control) and mycelium treated with voriconazole, fluconazole, or caspofungin alone exhibited a steady increase in metabolic activity, plateauing by t-9 (Fig 12A, B). In contrast, mycelium treated with CAP alone, amphotericin B alone, or any antifungal drug in combination with CAP exhibited a significant decrease in metabolic activity by t-9 (Fig 12B). This metabolic suppression was accompanied by a decrease in biofilm thickness in all CAP-treated groups (alone or with antifungals), as well as amphotericin B alone, with significant thinning observed within 3 h post-treatment (Fig 13A). In contrast, untreated controls and biofilms treated with voriconazole, fluconazole, or caspofungin alone did not change in thickness over the 24 h period, except for a slight but significant thickness reduction of voriconazole-treated biofilms between t-0 and t-24 (Fig 13B).

By t-9, each antifungal + CAP combination exhibited significantly lower metabolic activity than both the corresponding antifungal-only treatment and the untreated control, except for amphotericin B, where no significant difference was observed between drug-only and drug + CAP treatment (Fig 12D). While metabolic activity in CAP-only treated mycelium recovered by t-24 to levels indistinguishable from untreated controls (Fig 12E), biofilm thickness in CAP-treated groups remained significantly reduced for the 24 h period (Fig 13B). Notably, all antifungal and CAP-treated mycelium remained metabolically suppressed at t-24 relative to both untreated controls and CAP-only treatment. Among the combinations, only voriconazole + CAP showed partial metabolic recovery between t-9 and t-24; fluconazole + CAP, caspofungin + CAP, and amphotericin B (alone or with CAP) remained unchanged.

Compared to *A. flavus*, *F. keratoplasticum* demonstrated a slower and less complete recovery following CAP-treatment. Metabolic activity suppression in *F. keratoplasticum* persisted longer and was more resistant to recovery, particularly in the context of combined CAP and antifungal drug treatment across all drugs tested. In addition, CAP-mediated thinning of *F. keratoplasticum* biofilms was sustained across all treatment groups, unlike in *A. flavus*, where most biofilms increased in thickness by t-24.

### 3.4. Microscopic evaluation of the effects of CAP treatment on *A. flavus* and *F. keratoplasticum* mature mycelia

**Fig 14:**
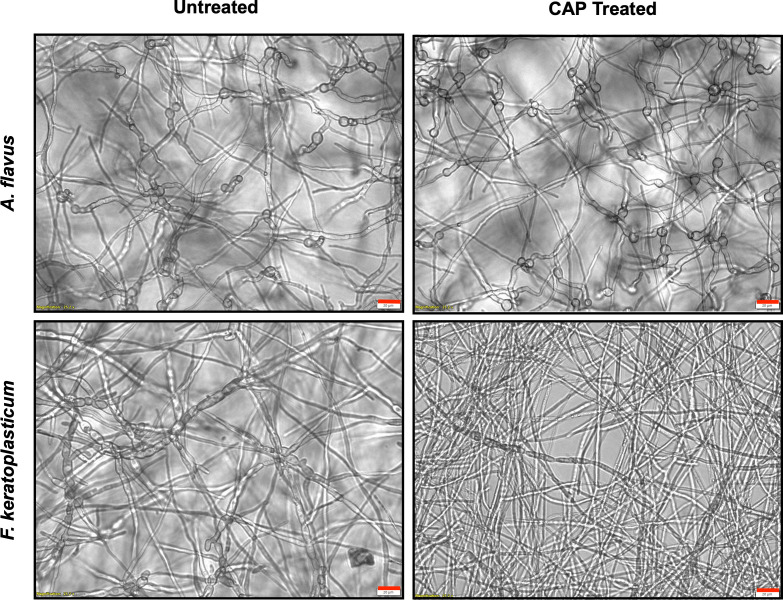
CAP treatment induces hyphal narrowing and loss of intracellular features in *F. keratoplasticum* mycelium, but not *A. flavus.* Brightfield microscopy (40X) demonstrating morphologic changes to *A. flavus* and *F. keratoplasticum* mycelium following CAP treatment (22 kV, 60 s) at 24 h post treatment compared with untreated controls. Scale bar (red) = 20 µm.

Brightfield microscopy was performed on CAP treated and untreated *A. flavus* and *F. keratoplasticum* mycelia 24 h after CAP treatment to assess for visible changes to hyphal organization and structure (Fig 14). CAP-treated *A. flavus* hyphae were not significantly altered based on microscopic examination compared to untreated *A. flavus*. In contrast, CAP-treated *F. keratoplasticum* displayed substantial morphologic changes with reduced hyphal density in CAP-treated biofilms. Hyphal width was also reduced following CAP treatment of *F. keratoplasticum*, coupled with fragmentation of hyphae.

## 4. Discussion

This study demonstrates that sublethal doses of cold atmospheric plasma (CAP) enhance antifungal drug susceptibility in *Aspergillus flavus* and *Fusarium keratoplasticum*, with effects that vary by fungal species, antifungal drug class (mode of action), and fungal growth development phase. CAP generally increased susceptibility in conidia, restoring fluconazole activity in *F. keratoplasticum* and *F. falciforme*, and increasing response to voriconazole and caspofungin in *A. flavus*. Among *F. falciforme* isolates, responses to combined treatments of CAP and fluconazole differed substantially, underscoring the importance of understanding and documenting species complex and strain-level genetic diversity.

We interpreted enhanced antifungal susceptibility as a greater reduction in CFU/mL with combined CAP and antifungal treatment than with either treatment alone. One example of this was observed in *F. keratoplasticum* conidia exposed to fluconazole, where the combination of fluconazole and CAP produced a significantly greater reduction in colony formation than either treatment individually. While the mechanisms underlying this response were not directly investigated here, previous studies in yeast and filamentous fungi have shown that CAP can increase membrane permeability and disrupt redox balance, potentially facilitating drug uptake or altering target accessibility [22,34–37]. These effects may contribute to the enhanced antifungal activity observed in some treatment contexts. In contrast, for combinations such as *A. flavus* with voriconazole or amphotericin B, where both treatments individually produced partial effects, the observed reductions in CFU/mL may reflect additive rather than sensitizing interactions.

While CAP typically enhanced antifungal efficacy in conidia, *A. flavus* exposed to amphotericin B exhibited an atypical response. MIC values increased following CAP treatment in liquid medium, and there was little visible difference in growth between treatment groups at low antifungal concentrations. However, CFU enumeration on agar revealed a paradoxical reduction in viability. One possible explanation is that CAP-induced lipid peroxidation alters membrane sterol composition, reducing amphotericin B binding to ergosterol. In liquid medium, this may appear as decreased drug efficacy, whereas CAP-treated conidia plated on agar may exhibit impaired colony formation despite prior growth potential. Notably, this response was not observed in *F. keratoplasticum*, suggesting that species-specific differences in membrane composition or stress adaptation may underlie divergent CAP–antifungal interactions that warrant further investigation.

In addition to conidial responses, mature biofilms exhibited comparable CAP-enhanced antifungal effects, with both metabolic activity and biofilm thickness showing reductions that varied by species and drug. In *F. keratoplasticum*, these effects were more sustained, whereas *A. flavus* biofilms exhibited partial recovery over time. CAP-induced metabolic suppression was evident within three hours, but by 24 h had recovered to be insignificantly different from untreated controls. Although combined CAP and antifungal drug treatments initially enhanced metabolic suppression in *A. flavus*, only voriconazole + CAP and amphotericin B + CAP produced significant reductions in mycelial viability and biofilm thickness at 24 hours. These results highlight a potential role for CAP in disrupting biofilm structure and reducing fungal viability, particularly in *F. keratoplasticum*, which is often resistant to commonly used antifungal drugs in clinical settings [32,33].

These findings support and extend previous reports in yeast and bacteria. Sublethal CAP exposure has been shown to reduce the MICs of fluconazole, amphotericin B, and caspofungin in *Candida albicans* biofilms by 2- to 6-fold, and to decrease the minimum biofilm eradication concentration (MBEC) of *Pseudomonas aeruginosa* biofilms by up to 128-fold when combined with tobramycin [22,26]. Our results demonstrate similar sensitizing effects in filamentous fungal pathogens. However, not all studies have observed enhanced efficacy from CAP-antifungal combinations. For example, Leite et al. reported that CAP alone was more effective than CAP plus amphotericin B or nystatin in reducing *Candida albicans* CFU/mL [34]. Such discrepancies likely reflect differences in fungal species, developmental growth stage, and experimental design, and highlight the need for broader comparative studies across diverse pathogenic microbes.

Although our study did not directly measure plasma-generated reactive species, the antifungal effects of CAP have been linked to the generation of short- and long-lived reactive oxygen and nitrogen species (RONS), including superoxide anion, hydrogen peroxide, nitric oxide, and peroxynitrite. These species can induce oxidative damage to membrane lipids, proteins, and nucleic acids, and have been shown to alter fungal membrane permeability, disrupt ion homeostasis, and damage cell wall polysaccharides such as chitin and β-glucan [35–37]. Such changes are likely to increase intracellular penetration or alter drug-target interactions, thereby contributing to the increased antifungal effect observed following CAP treatment. In this context, the restoration of fluconazole responsiveness in *Fusarium* species, for example, may reflect improved drug uptake following CAP-induced membrane disruption. Further studies measuring reactive species and correlating them with structural or biochemical changes in fungal cells will be important for confirming the mechanistic basis of this enhanced antifungal response.

Taken together, these findings suggest that while CAP alone does not produce sustained metabolic suppression, its combination with antifungal drugs can enhance persistence of antifungal activity. The rapid but transient increase in metabolic activity observed following CAP treatment of *F. keratoplasticum* mycelium may represent a stress-induced response that antifungal drugs capitalize on to prevent recovery. Identifying the timing of maximal viability suppression and onset of recovery may have clinical implications. This temporal window may offer an opportunity for repeated CAP treatments to be administered before fungal growth resumes. Reapplication of CAP during this phase could potentially increase cumulative antifungal efficacy by targeting metabolically stressed cells before compensatory mechanisms are activated. Imaging of CAP-treated *F. keratoplasticum* mycelium revealed hyphal narrowing consistent with membrane damage that may lead to intracellular leakage and loss of viability. These observations support previous reports describing CAP-induced membrane permeability changes and cytosolic leakage in fungal conidia and biofilms [35–37]. The sustained suppression of both metabolic activity and biofilm thickness observed with combination treatment, particularly in *F. keratoplasticum*, suggests that CAP enhances antifungal efficacy by compromising fungal structure and limiting regrowth. Future studies should investigate whether repeated CAP exposure, timed to intercept early recovery fungal growth phases, can achieve more durable suppression or prevent recovery altogether.

To our knowledge, this work represents the first comprehensive assessment of CAP’s effects on antifungal response in filamentous fungi, including both planktonic and biofilm phases. A key strength is the inclusion of multiple antifungal classes, enabling a broad evaluation of CAP’s interaction with distinct drug mechanisms. Using both MIC assays and CFU enumeration further strengthened our findings by providing complementary assessments of fungal viability. However, there are several limitations using this experimental approach. All experiments were performed *in vitro*, and *in vivo* studies are needed to assess therapeutic potential. Biofilm susceptibility was evaluated using a single antifungal concentration based on conidial MICs, limiting insights into dose-dependent effects. CFU enumeration in a biofilm context remains technically challenging and may underestimate viable but non-culturable cells. Additionally, although CAP was demonstrated to broadly enhance antifungal activity, synergistic effects were not formally assessed and should be addressed in future work. Finally, broader isolate-level comparisons, particularly within the *Fusarium* and *Aspergillus* species complexes, are necessary to determine the extent to which these findings are conserved or species-specific. Additional work is needed to assess how consistently CAP modifies antifungal responses across genetically and phenotypically diverse clinical isolates

In conclusion, sublethal CAP enhances antifungal susceptibility in *A. flavus*, *F. keratoplasticum*, and *F. falciforme*, though effects are species- and drug-dependent. CAP restored antifungal response to fluconazole and caspofungin activity in *F. keratoplasticum*, a species with reported resistance to multiple antifungal classes. Similarly, three strains of *F. falciforme* belonging to the same species complex and multi-locus haplotype demonstrated a range of responses, with some showing marked improvement in fluconazole susceptibility following CAP treatment and others exhibiting inherent CAP sensitivity. The atypical interaction between CAP and amphotericin B in *A. flavus* underscores the complexity of CAP-drug interactions and need for mechanistic clarity. Importantly, CAP also demonstrated additive effects in reducing biofilm viability and thickness, particularly in *F. keratoplasticum*, suggesting it may be an effective adjunct for targeting biofilm-associated infections. These findings support continued exploration of CAP as a promising antifungal adjuvant. Combined treatment with CAP and antifungal drugs could be integrated into fungal keratitis management as an adjunct to topical antifungal therapy, particularly in cases involving drug-resistant organisms or biofilm-associated infections. In practice, CAP could be applied to the infected cornea at the time of diagnosis or during early treatment to reduce fungal viability, enhance drug responsiveness, and improve drug access by disrupting biofilm structure. This may help accelerate resolution, reduce treatment duration, and potentially lower the need for surgical intervention. However, this approach is not without limitations. The duration of CAP’s antifungal effects appears transient in some cases, suggesting that repeated exposure may be necessary. Additionally, fungal response varies by species, strain, and drug class, requiring further optimization of treatment protocols. While initial ex vivo and in vivo studies have shown CAP to be well tolerated, comprehensive safety and efficacy trials in clinically infected eyes are still needed to fully define its therapeutic role.

## Supporting information

S1 Fig Representative phase-contrast images of *Aspergillus flavus* biofilms at t-0 and t-24 from the metabolic activity assay. Representative images of *A. flavus* biofilms acquired at t-0 and t-24 during the XTT-based metabolic activity assay. Each treatment group is shown: untreated, CAP alone, antifungal alone, and CAP + antifungal (voriconazole, fluconazole, amphotericin B, or caspofungin. Images were used in pixel-based analysis to quantify metabolically active mycelial area. Image magnification: 2.52x. Scale bar = 200 µm.
(TIF)

S2 Fig Representative phase-contrast images of *Fusarium keratoplasticum* biofilms at t-0 and t-24 from the metabolic activity assay. Representative images of *F. keratoplasticum* biofilms acquired at t-0 and t-24 during the XTT-based metabolic activity assay. Each treatment group is shown: untreated, CAP alone, antifungal alone, and CAP + antifungal (voriconazole, fluconazole, amphotericin B, or caspofungin. Images were used in pixel-based analysis to quantify metabolically active mycelial area. Image magnification: 2.52x. Scale bar = 200 µm.
(TIF)

S1 Table P-values from comparisons between antifungal drug-treated and untreated conidial samples in CAP-treated and untreated groups. Two-way Analysis of Variance (ANOVA) with Šídák’s multiple comparisons test. Each p-value reflects a comparison between the specified antifungal dose and the 0 µg/mL untreated control within the same CAP treatment condition. P-values < 0.05 were considered statistically significant.
(DOCX)
