# Supplementary figures and images for: Cold atmospheric plasma improves antifungal responsiveness of Aspergillus flavus and Fusarium keratoplasticum conidia and mycelia
Source: PLoS One. 2025 Aug 11;20(8):e0326940. doi: 10.1371/journal.pone.0326940 (PMC12338820; doi:10.1371/journal.pone.0326940)

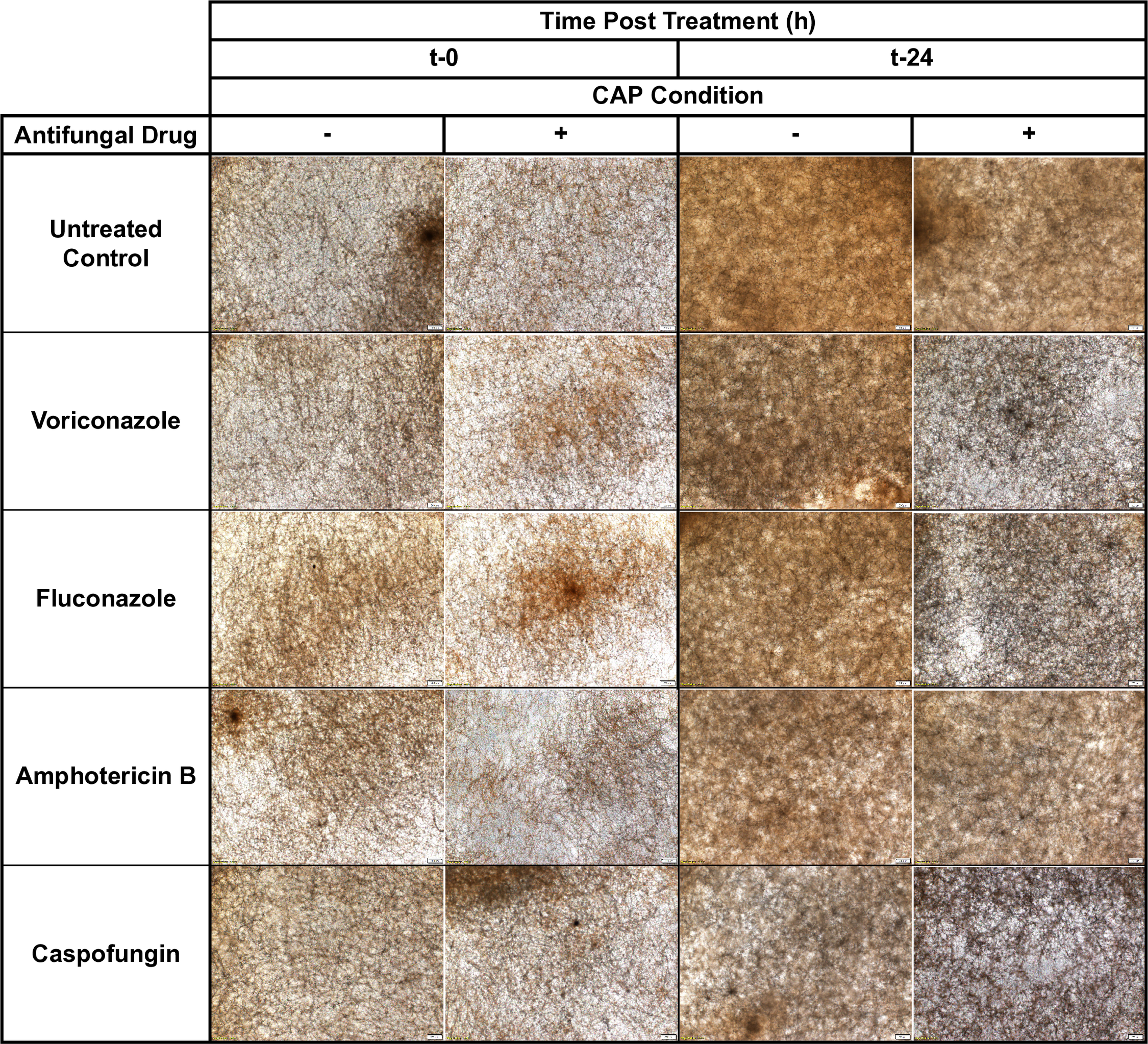

Supplement: S1 Fig — Representative images of A. flavus biofilms acquired at t-0 and t-24 during the XTT-based metabolic activity assay. Each treatment group is shown: untreated, CAP alone, antifungal alone, and CAP + antifungal (voriconazole, fluconazole, amphotericin B, or caspofungin. Images were used in pixel-based analysis to quantify metabolically active mycelial area. Image magnification: 2.52x. Scale bar = 200 µm. (TIF) [file pone.0326940.s001.tif]

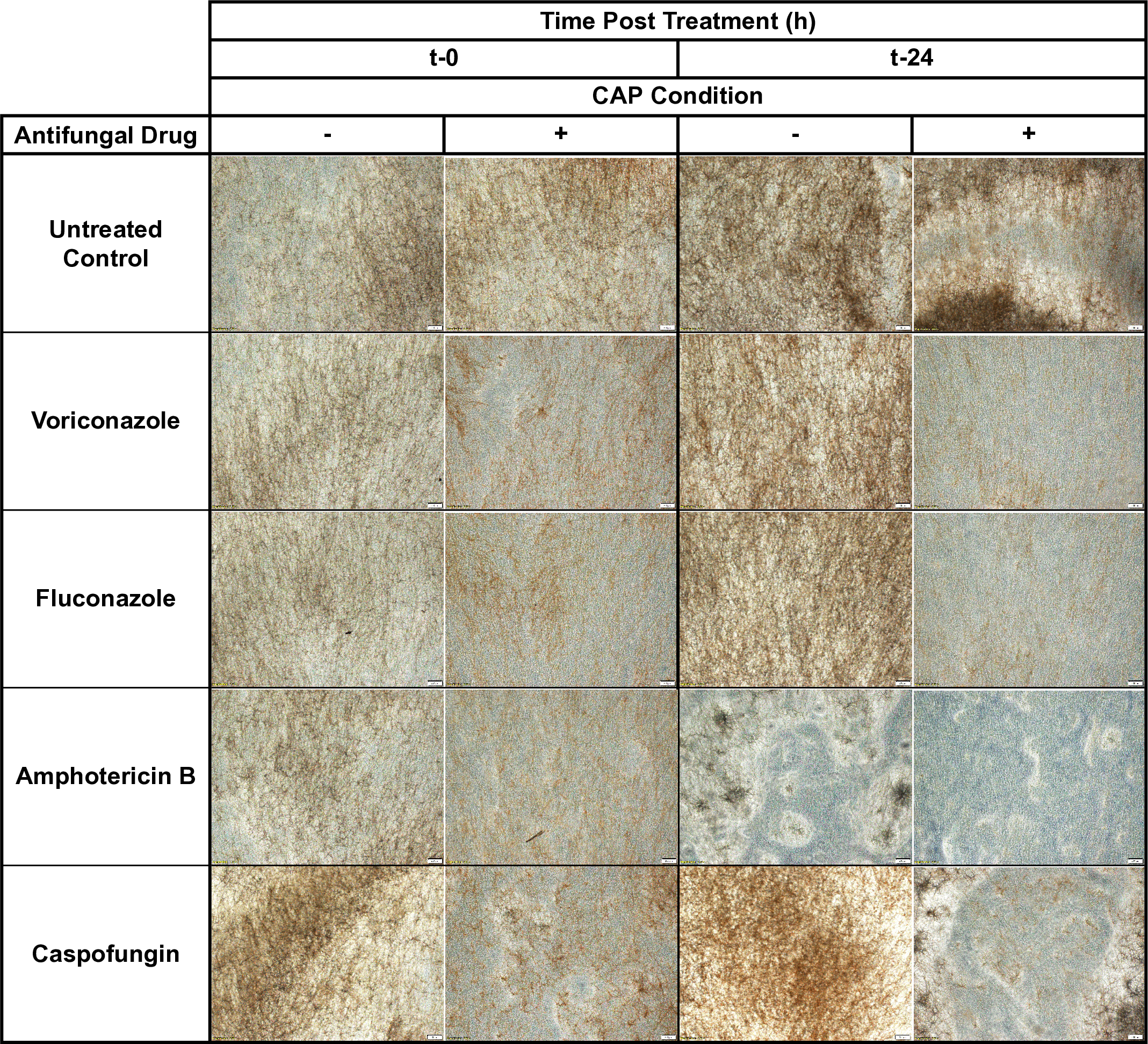

Supplement: S2 Fig — Representative images of F. keratoplasticum biofilms acquired at t-0 and t-24 during the XTT-based metabolic activity assay. Each treatment group is shown: untreated, CAP alone, antifungal alone, and CAP + antifungal (voriconazole, fluconazole, amphotericin B, or caspofungin. Images were used in pixel-based analysis to quantify metabolically active mycelial area. Image magnification: 2.52x. Scale bar = 200 µm. (TIF) [file pone.0326940.s002.tif]
