# Supplementary material for: Cold atmospheric plasma improves antifungal responsiveness of Aspergillus flavus and Fusarium keratoplasticum conidia and mycelia
Source: PLoS One. 2025 Aug 11;20(8):e0326940. doi: 10.1371/journal.pone.0326940 (PMC12338820; doi:10.1371/journal.pone.0326940)
Supplement: S1 Table — Two-way Analysis of Variance (ANOVA) with Šídák’s multiple comparisons test. Each p-value reflects a comparison between the specified antifungal dose and the 0 µg/mL untreated control within the same CAP treatment condition. P-values < 0.05 were considered statistically significant. (DOCX) [file pone.0326940.s003.docx]

| **Fungal Species** | **Antifungal Drug** | **CAP Condition** | **Dose (μg/mL)** | **p-value vs. 0 µg/mL Control** | **Figure Reference** |
| --- | --- | --- | --- | --- | --- |
| *Aspergillus flavus* | Voriconazole | Untreated | 0.0078 | >0.9999 | Fig 1 |
|  |  |  | 0.0156 | 0.9347 |  |
|  |  |  | 0.03125 | <0.0001 |  |
|  |  |  | 0.0625 | <0.0001 |  |
|  |  |  | 0.125 | <0.0001 |  |
|  |  |  | 0.25 | <0.0001 |  |
|  |  | Treated | 0.0078 | 0.0935 |  |
|  |  |  | 0.0156 | <0.0001 |  |
|  |  |  | 0.03125 | <0.0001 |  |
|  |  |  | 0.0625 | <0.0001 |  |
|  |  |  | 0.125 | <0.0001 |  |
|  |  |  | 0.25 | <0.0001 |  |
|  | Fluconazole | Untreated | 4.875 | 0.7498 | Fig 2 |
|  |  |  | 9.75 | 0.0495 |  |
|  |  |  | 19.5 | <0.0001 |  |
|  |  |  | 39 | <0.0001 |  |
|  |  |  | 78 | <0.0001 |  |
|  |  |  | 156 | <0.0001 |  |
|  |  | Treated | 4.875 | >0.9999 |  |
|  |  |  | 9.75 | 0.9344 |  |
|  |  |  | 19.5 | 0.6133 |  |
|  |  |  | 39 | 0.3153 |  |
|  |  |  | 78 | 0.0059 |  |
|  |  |  | 156 | 0.0008 |  |
|  | Amphotericin B | Untreated | 0.125 | 0.3316 | Fig 3 |
|  |  |  | 0.25 | 0.0017 |  |
|  |  |  | 0.5 | 0.0003 |  |
|  |  |  | 1 | <0.0001 |  |
|  |  |  | 2 | <0.0001 |  |
|  |  |  | 4 | <0.0001 |  |
|  |  | Treated | 0.125 | >0.9999 |  |
|  |  |  | 0.25 | >0.9999 |  |
|  |  |  | 0.5 | 0.9888 |  |
|  |  |  | 1 | 0.7770 |  |
|  |  |  | 2 | 0.9041 |  |
|  |  |  | 4 | 0.4905 |  |
|  | Caspofungin | Untreated | 4.875 | <0.0001 | Fig 4 |
|  |  |  | 9.75 | <0.0001 |  |
|  |  |  | 19.5 | <0.0001 |  |
|  |  |  | 39 | <0.0001 |  |
|  |  |  | 78 | <0.0001 |  |
|  |  |  | 156 | <0.0001 |  |
|  |  | Treated | 4.875 | <0.0001 |  |
|  |  |  | 9.75 | <0.0001 |  |
|  |  |  | 19.5 | <0.0001 |  |
|  |  |  | 39 | <0.0001 |  |
|  |  |  | 78 | <0.0001 |  |
|  |  |  | 156 | <0.0001 |  |
| *Fusarium keratoplasticum* | Voriconazole | Untreated | 0.25 | 0.0235 | Fig 5 |
|  |  |  | 0.5 | <0.0001 |  |
|  |  |  | 1 | <0.0001 |  |
|  |  |  | 2 | <0.0001 |  |
|  |  |  | 4 | <0.0001 |  |
|  |  |  | 8 | <0.0001 |  |
|  |  | Treated | 0.25 | 0.0060 |  |
|  |  |  | 0.5 | <0.0001 |  |
|  |  |  | 1 | <0.0001 |  |
|  |  |  | 2 | <0.0001 |  |
|  |  |  | 4 | <0.0001 |  |
|  |  |  | 8 | <0.0001 |  |
|  | Fluconazole | Untreated | 4.875 | >0.9999 | Fig 6 |
|  |  |  | 9.75 | 0.9539 |  |
|  |  |  | 19.5 | 0.9616 |  |
|  |  |  | 39 | 0.9970 |  |
|  |  |  | 78 | 0.8442 |  |
|  |  |  | 156 | 0.9946 |  |
|  |  | Treated | 4.875 | 0.9126 |  |
|  |  |  | 9.75 | 0.2054 |  |
|  |  |  | 19.5 | 0.3403 |  |
|  |  |  | 39 | 0.0445 |  |
|  |  |  | 78 | 0.0082 |  |
|  |  |  | 156 | 0.0195 |  |
|  | Amphotericin B | Untreated | 4.875 | 0.0351 | Fig 7 |
|  |  |  | 9.75 | 0.0001 |  |
|  |  |  | 19.5 | <0.0001 |  |
|  |  |  | 39 | <0.0001 |  |
|  |  |  | 78 | <0.0001 |  |
|  |  |  | 156 | <0.0001 |  |
|  |  | Treated | 4.875 | 0.9024 |  |
|  |  |  | 9.75 | 0.8278 |  |
|  |  |  | 19.5 | 0.0006 |  |
|  |  |  | 39 | 0.0046 |  |
|  |  |  | 78 | <0.0001 |  |
|  |  |  | 156 | <0.0001 |  |
|  | Caspofungin | Untreated | 0.5 | >0.9999 | Fig 8 |
|  |  |  | 1 | 0.0045 |  |
|  |  |  | 2 | <0.0001 |  |
|  |  |  | 4 | <0.0001 |  |
|  |  |  | 8 | <0.0001 |  |
|  |  |  | 16 | <0.0001 |  |
|  |  | Treated | 0.5 | 0.7009 |  |
|  |  |  | 1 | 0.0073 |  |
|  |  |  | 2 | <0.0001 |  |
|  |  |  | 4 | <0.0001 |  |
|  |  |  | 8 | <0.0001 |  |
|  |  |  | 16 | <0.0001 |  |
| *F. falciforme*  IC22919 | Fluconazole | Untreated | 4.875 | >0.9999 | Fig 9A |
|  |  |  | 9.75 | 0.9845 |  |
|  |  |  | 19.5 | 0.9995 |  |
|  |  |  | 39 | >0.9999 |  |
|  |  |  | 78 | >0.9999 |  |
|  |  |  | 156 | 0.7869 |  |
|  |  | Treated | 4.875 | 0.0018 |  |
|  |  |  | 9.75 | 0.0023 |  |
|  |  |  | 19.5 | 0.0075 |  |
|  |  |  | 39 | 0.1326 |  |
|  |  |  | 78 | 0.0024 |  |
|  |  |  | 156 | 0.0001 |  |
| *F. falciforme*  IC22927 | Fluconazole | Untreated | 4.875 | 0.4517 | Fig 9B |
|  |  |  | 9.75 | 0.0868 |  |
|  |  |  | 19.5 | 0.0808 |  |
|  |  |  | 39 | 0.0626 |  |
|  |  |  | 78 | 0.0187 |  |
|  |  |  | 156 | <0.0001 |  |
|  |  | Treated | 4.875 | 0.1579 |  |
|  |  |  | 9.75 | 0.0002 |  |
|  |  |  | 19.5 | 0.0002 |  |
|  |  |  | 39 | 0.0008 |  |
|  |  |  | 78 | <0.0001 |  |
|  |  |  | 156 | <0.0001 |  |
| *F. falciforme*  IC22918 | Fluconazole | Untreated | 4.875 | 0.0186 | Fig 9C |
|  |  |  | 9.75 | 0.0703 |  |
|  |  |  | 19.5 | 0.0669 |  |
|  |  |  | 39 | <0.0001 |  |
|  |  |  | 78 | 0.0007 |  |
|  |  |  | 156 | <0.0001 |  |
|  |  | Treated | 4.875 | 0.2559 |  |
|  |  |  | 9.75 | 0.7393 |  |
|  |  |  | 19.5 | 0.6346 |  |
|  |  |  | 39 | 0.9431 |  |
|  |  |  | 78 | 0.6178 |  |
|  |  |  | 156 | 0.2276 |  |
